# Leiomyosarcoma of Pulmonary Vein Presenting as Left Atrial Mass: An Outline of Management Principles

**DOI:** 10.1155/2012/396319

**Published:** 2012-10-24

**Authors:** Philemon Gukop, Guido Frassetto, Georgios Karapanagiotidis, Venkatachalam Chandrasekaran

**Affiliations:** Department of Cardiothoracic Surgery, St George's Hospital, London SW17 0QT, UK

## Abstract

Leiomyosarcoma of the pulmonary vein is rare and has poor prognosis. Its clinical features are nonspecific and mimic benign conditions. Early diagnosis is challenging. Most cases have been diagnosed only at autopsy or on postoperative histology specimens. Treatment is essentially palliative complete surgical excision. We outline the principles of management with the case of a 39-year-old man with leiomyosarcoma of the left pulmonary veins extending into the left atrium. Extensive investigation to achieve early diagnosis and determine extent of disease is essential. Frozen section guided adequate excision of all cardiac tumours and resection of involved lung tissue achieve local disease control. Adjuvant chemoradiotherapy has been shown to enhance survival.

## 1. Case Report

**Figure 1:**
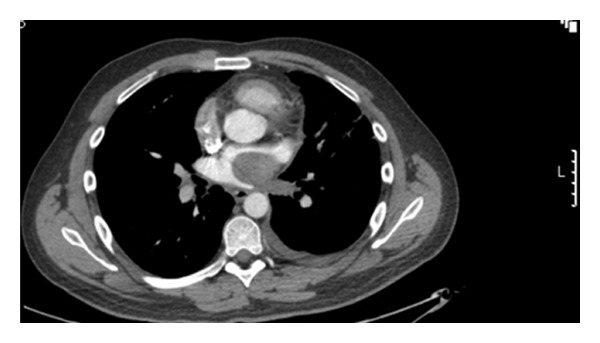
Computer tomogram showing left atrial mass extending into left pulmonary veins.

A 39-year-old gentleman presented with recurrent shortness of breath, episodes of desaturation, chest pain, and haemoptysis. He was treated for recurrent chest infection. Computerised tomography scan showed a well circumscribed 4 cm diameter mass within the left atrium with some abnormal tissue within the left sided pulmonary veins which showed interval increase in size (Figure 1). 

**Figure 2:**
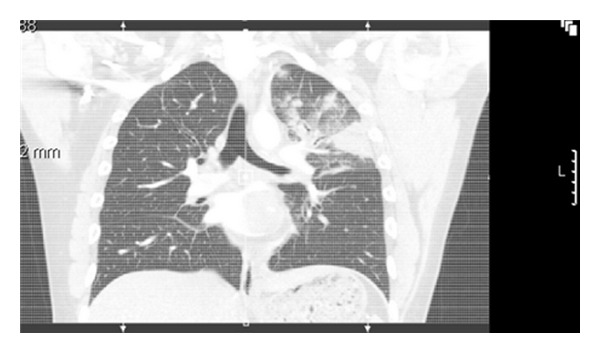
Computer tomogram of chest showing left atrial mass extending into pulmonary vein with left upper lobe lung consolidation.

There was a multifocal ground glass opacity, nodularity, and consolidative changes within the left upper lobe representing a combination of infection, haemorrhage, and infarction (Figure 2). 

**Figure 3:**
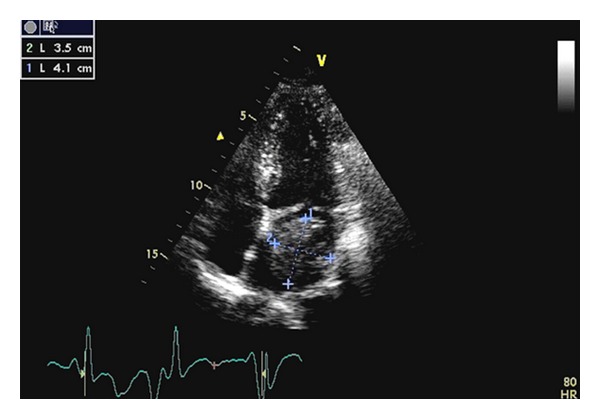
Transthoracic echocardiogram showing left atrial mass obstructing left ventricular outlet tract and originating from left superior pulmonary vein.

Echocardiogram confirmed a large left atrial mass measuring 3.6 by 4.5 cm causing mitral valve obstruction and appears to originate from the left superior pulmonary vein (Figure 3).

**Figure 4:**
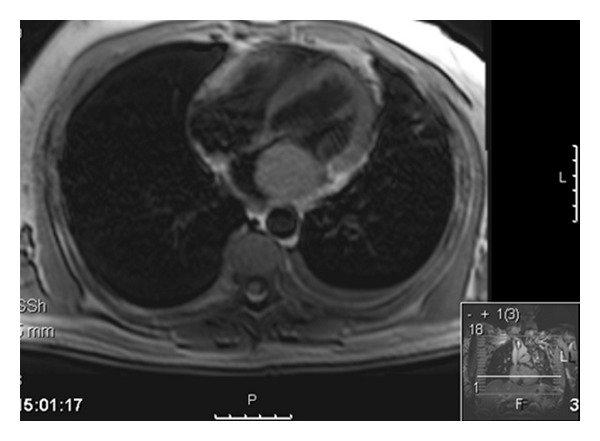
Cardiac magnetic resonance scan (MRI) showing left atrial mass obstructing left ventricular outlet tract.

Cardiac magnetic resonance imaging (MRI) showed a large (44 mm × 41 mm HLA) mass in the left atrium that appears to arise from the left upper lobe pulmonary vein and is not attached to the atrial septum (Figure 4). Following gadolinium there was heterogeneous opacification of the mass with absent filling of the left pulmonary veins.

Median sternotomy, aortic-bicaval cannulation, and standard cardiopulmonary bypass were performed for excision of the left atrial mass. Via a right interatrial grove incision a large pedunculated, fibrous, hard mass with smooth surface occupying two thirds of the left atrium was excised; the pedicle was traced to the left superior pulmonary vein. The mass was adherent to the atrial wall by fibrous tissue which was released. It extended into the left superior pulmonary vein and totally occluding the lumen; the vein felt hard from the outside up into its proximal branches. Similar tissues extending into the right pulmonary veins were excised in the fashion of an endarterectomy. The inferior left pulmonary vein felt normal.

Frozen section of biopsies from the atrial mass and left upper lobe lesion suggested malignancy. A left upper lobectomy was performed.

Postoperative recovery was uneventful. Histology of the atrial mass showed interlacing bundles of spindle cells with eosinophilic cytoplasm and cigar shaped nuclei; in some areas of tumour sampled there was focal severe pleomorphism with rhabdoid cells and multinucleate tumour giant cells and focal necrosis. The mitotic rate was variable but in the most pleomophic area it was 12 per 10 high power field. The tumour cells were desmin and actin positive, MyoD1 positive both in nuclei and cytoplasm. EMA, caldesmon, and S100 were negative. The appearances were those of high grade leiomyosarcoma with incompletely excised atrial margin. 

Histological sections from the lung resection showed high grade leiomyosarcoma involving the left pulmonary vein extending into the lumen and also into the left atrium. The tumour infiltrates the vein wall and is present on the vein wall surface in several sections but no evidence of invasion of lung parenchyma. The distal lung showed local extension of tumour along branches of pulmonary vein. Sampled lymph nodes showed no evidence of malignancy. He had adjuvant radiotherapy/chemotherapy and at 6-month followup there was wide spread metastasis involving the bones; no local recurrence was noted.

## 2. Discussion

Leiomyosarcoma of the pulmonary vein is a rare pathology with an incidence of 0.25% and a poor prognosis [1]. Survival has been estimated to about 6–12 months without surgery [1]. Up to 60% of cases have metastasis at the time of diagnosis and microembolisation is common [2]. Metastasis has been described as a natural course of leiomyosarcoma even after radical surgical excision and the average time to local recurrence is about 6 months [3]. Clinical presentation is nonspecific; this makes early diagnosis difficult and most cases are diagnosed on histology specimens and at autopsy [2]. It is twice as common in females; the average age of occurrence is 45 years ( range 6 weeks to 77 years) [4].

This 39-year-old man presented to the chest physicians with cough, chest pain, shortness of breath, and haemoptysis; initial chest radiograph showed a left upper lobe consolidation which persisted despite courses of antibiotics. Computerised tomogram suggested this to be an infective or infarctive lesion with some haemorrhage; it further revealed a filling defect in the left superior pulmonary vein with a left atrial mass arising from the left pulmonary vein. Similar nonspecific symptoms have been reported in other cases of leiomyosarcoma of the pulmonary vein [5]. Magnetic resonant imaging (MRI) has been shown to differentiate the tumour from a thrombus by contrast enhancement with gadolinium labelled diethylene pentamine triacetic acid [5]. The symptoms of this aggressive condition mimic those of benign conditions like chest infection, myxoma, and pulmonary thromboembolism. A nonresolving chest infection or suspected pulmonary thromboembolism that does not resolve or progresses despite adequate treatment should arouse the suspicion of leiomyosarcoma and further investigation by computerised tomography and gadolinium contrast MRI scan to achieve early diagnosis and treatment [6]. Transvenous catheter suction biopsy [7] and fine needle aspiration biopsy of pulmonary vein leiomyosarcoma have been reported [8]. These techniques can be used to achieve preoperative diagnosis. A left atrial mass is traditionally regarded as a benign myxoma [9]. Out of 1000 cardiac tumours biopsied 77% were myxoma and 10% were sarcomas. Sarcomas mostly occur on the right side of the heart while myxoma occurs predominantly in the left atrium. The exception to this rule is leiomyosarcoma which mostly occurs in the left atrium and may mimic a myxoma especially the myxoid type of leiomyosarcoma [10]. It is critical to exclude leiomyosarcoma as a differential diagnosis of all left atrial mass by histology, as this has prognostic and management significance in terms of margin of excision and the use of adjuvant chemotherapy and/or radiotherapy. 

Early diagnosis and adequate surgical resection is the main stay of treatment of pulmonary vein leiomyosarcoma. This is palliative, as it runs a progressive course despite adequate resection. Adjuvant radiotherapy and chemotherapy have been shown to prolong survival and enhance disease control in some cases [10]. Morin and colleagues suggested frozen section guided excision margin of 1cm for all left atrial tumours for better survival [10]. Adequate resection of pulmonary vein tumour may require lung resection ranging from lobectomy to pneumonectomy. Our case demonstrates that involvement of pulmonary veins usually extend to smaller intraparenchymal branches of the vein. 

Heart transplantation is advocated as an option in suitable cases [11]. Leiomyosarcoma is known to express oestrogen and progesterone receptors; this may have therapeutic implication for hormone modulation [12]. 

Doxorubicin, ifosfamide, vincristine, etoposide, uracil, cisplatin, cyclophosphamide, and mitomycin are amongst chemotherapeutic agents that have been tried, with varied outcomes. Leiomyosarcoma has low radiosensitivity and high dose radiation could cause myocarditis and pericarditis [1].

Factors which determine survival include mitotic index of the tumour, presence of metastasis, necrosis in tumour section, and localisation of tumour to left side of the heart [1].

## 3. Conclusion

Leiomyosarcoma of the pulmonary vein has poor prognosis; treatment is palliative frozen section guided complete surgical excision. Adjuvant chemoradiotherapy may further prolong survival. Its clinical features and treatment are still inadequately understood; further research and reporting of this cases should be encouraged.
